# 5,5′-[(1,4-Phenyl­enedimethyl­ene)bis­(sulfanedi­yl)]bis­(1-methyl-1*H*-1,2,3,4-tetra­zole)

**DOI:** 10.1107/S1600536811043182

**Published:** 2011-10-29

**Authors:** Dan-Feng He, Jin-Jun Deng, Fu-Jiang Zhou, Hong-Sheng Liu, Li-Min Wang

**Affiliations:** a Department of Chemistry and Chemical Engineering, Daqing Normal University, 163712 Daqing, Heilongjiang, People’s Republic of China; b DaQing Petrochemical Corporation, 163712 Daqing, Heilongjiang, People’s Republic of China

## Abstract

The title mol­ecule, C_12_H_14_N_8_S_2_, has point symmetry 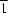 since it is situated on a crystallographic centre of symmetry. The 1-meth­yl/5-thio groups are in an anti­periplanar conformation. The dihedral angle between the benzene and tetra­zole rings is 84.33 (2)°. In the crystal, C—H⋯N hydrogen bonds link mol­ecules into ladder-like chains running along the *b* axis. There are also C—H⋯π inter­actions present in the crystal structure.

## Related literature

For the pharmaceutical properties of ligands derived from tetra­zole, see: Armour *et al.* (1996 ▶); Segarra *et al.* (1998 ▶); Bronisz (2002 ▶); Semenov (2002 ▶); Upadhayaya *et al.* (2004 ▶); Wang *et al.* (2004 ▶); She *et al.* (2006 ▶); Wei *et al.* (2011 ▶). For the synthesis of the title compound, see: Wang *et al.* (2005 ▶). For graph-set motifs, see: Etter *et al.* (1990 ▶).
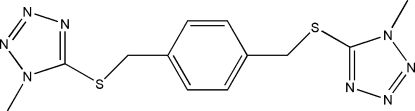


## Experimental

### 

#### Crystal data

C_12_H_14_N_8_S_2_
*M* *_r_* = 334.43
Monoclinic, 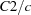

*a* = 18.464 (4) Å
*b* = 7.6392 (18) Å
*c* = 13.625 (3) Å
β = 126.999 (4)°
*V* = 1534.8 (6) Å^3^
*Z* = 4
Mo *K*α radiation
μ = 0.36 mm^−1^
*T* = 296 K
0.25 × 0.10 × 0.10 mm

#### Data collection

Bruker SMART APEXII diffractometer
Absorption correction: multi-scan (*SADABS*; Bruker, 2005 ▶) *T*_min_ = 0.916, *T*_max_ = 0.965
6541 measured reflections
1758 independent reflections
1412 reflections with *I* > 2σ(*I*)
*R*_int_ = 0.024

#### Refinement

*R*[*F* ^2^ > 2σ(*F* ^2^)] = 0.040
*wR*(*F* ^2^) = 0.116
*S* = 1.07
1758 reflections
101 parameters
H-atom parameters constrained
Δρ_max_ = 0.42 e Å^−3^
Δρ_min_ = −0.26 e Å^−3^

### 

Data collection: *APEX2* (Bruker, 2005 ▶); cell refinement: *SAINT* (Bruker, 2005 ▶); data reduction: *SAINT*; program(s) used to solve structure: *SHELXTL* (Sheldrick, 2008 ▶); program(s) used to refine structure: *SHELXTL*; molecular graphics: *SHELXTL*; software used to prepare material for publication: *SHELXTL*.

## Supplementary Material

Crystal structure: contains datablock(s) global, I. DOI: 10.1107/S1600536811043182/fb2238sup1.cif

Structure factors: contains datablock(s) I. DOI: 10.1107/S1600536811043182/fb2238Isup2.hkl

Supplementary material file. DOI: 10.1107/S1600536811043182/fb2238Isup3.cml

Additional supplementary materials:  crystallographic information; 3D view; checkCIF report

## Figures and Tables

**Table 1:** Hydrogen-bond geometry (Å, °) *Cg*_benzene_ is the centroid of the benzene ring.

*D*—H⋯*A*	*D*—H	H⋯*A*	*D*⋯*A*	*D*—H⋯*A*
C4—H4*B*⋯N2^i^	0.97	2.58	3.429 (3)	145
C6—H6*A*⋯*Cg*_benzene_^ii^	0.96	2.82	3.545 (4)	133

Symmetry codes: (i) 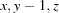; (ii) 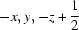.

**Fig. 1.**
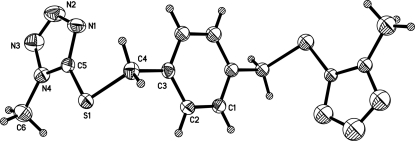
The title molecule with the displacement ellipsoids drawn at the 30% probability level.

**Fig. 2.**
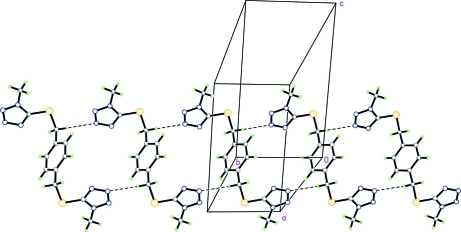
The infinite ladder-like chains formed via C-H···N hydrogen bonds indicated by the dashed lines. The chains contain the graph set motifs R22(22) (Etter et al., 1990).

## supplementary crystallographic information

### Comment

In recent years, tetrazole compounds and their derivatives have received much attention because of their diverse pharmaceutical properties (Armour *et al.*, 1996; Segarra *et al.*, 1998; Bronisz, 2002; Semenov, 2002; Upadhayaya *et al.*, 2004; Wang *et al.*, 2004; She *et al.*, 2006; Wei *et al.*, 2011).

In order to search for a new tetrazole compound with higher bioactivity, the title compound has been synthesized and its crystal structure determined.

The title molecule, C_12_H_14_N_2_S_8_, has the point symmetry 1 since it is situated on the crystallographic centre of symmetry. 1-methyl-5-thio- moieties are in the antiperiplanar conformation. The dihedral angle between the benzene and tetrazole ring is 84.39 (2) °. The molecules are situated on the crystallographic centres of symmetry and therefore their point symmetry is 1. In the crystal structure, the molecules are linked by C—H···N hydrogen bonds (Tab. 1; Fig. 2) forming a ladder-like chain composed of the graph set motifs *R*^2^_2_(22) (Etter *et al.* (1990). The chains are directed along the *b* axis.

Moreover, there are also C—H···ring-πi-electron interactions in the structure: (C6—H6A···*Cg*_benzene_^i^: 0.96, 2.82, 3.545 (4) Å, 133 °; the symmetry code i: -*x*, *y*, 1/2 - *z* and C6^ii^—H6A^ii^···*Cg*_benzene_^ii^: 0.96, 2.82, 3.545 (4) Å, 133 °; the symmetry code ii: *x*, -*y*, 1/2 + *z*).

### Experimental

The title compound was synthesized according to the method reported in the literature (Wang *et al.*, 2005). Colourless block-shaped crystals with approx. size 0.2 × 0.1 × 0.1 mm were obtained by slow evaporation from ethanol solution of the title compound.

### Refinement

All the H atoms could be discerned in the difference electron density map. However, they have been situated into the idealized positions and refined within the riding atom approximation. The used constraints: C_aryl_—H_aryl_ = 0.93; C_methyl_—H_methyl_ = 0.96 Å; C_methylene_—H_methylene_ = 0.97 Å. *U*_iso_(H_aryl_/_methylene_)=1.2; *U*_iso_(H_methyl_)=1.5*U*_eq_(C_methyl_). The diffraction 2 0 0 has been excluded from the refinement because most probably it had been eclipsed by the beam stop.

### Crystal data

**Table:**

C_12_H_14_N_8_S_2_	*F*(000) = 696
*M**_r_* = 334.43	*D*_x_ = 1.447 Mg m^−^^3^
Monoclinic, *C*2/*c*	Mo *K*α radiation, λ = 0.71073 Å
Hall symbol: -C 2yc	Cell parameters from 2201 reflections
*a* = 18.464 (4) Å	θ = 3.0–26.9°
*b* = 7.6392 (18) Å	µ = 0.36 mm^−^^1^
*c* = 13.625 (3) Å	*T* = 296 K
β = 126.999 (4)°	Block, colorless
*V* = 1534.8 (6) Å^3^	0.25 × 0.10 × 0.10 mm
*Z* = 4	

### Data collection

**Table:**

Bruker SMART APEXII diffractometer	1758 independent reflections
Radiation source: fine-focus sealed tube	1412 reflections with *I* > 2σ(*I*)
graphite	*R*_int_ = 0.024
φ and ω scans	θ_max_ = 27.6°, θ_min_ = 3.0°
Absorption correction: multi-scan (*SADABS*; Bruker, 2005)	*h* = −19→24
*T*_min_ = 0.916, *T*_max_ = 0.965	*k* = −9→9
6541 measured reflections	*l* = −17→15

### Refinement

**Table:**

Refinement on *F*^2^	Primary atom site location: structure-invariant direct methods
Least-squares matrix: full	Secondary atom site location: difference Fourier map
*R*[*F*^2^ > 2σ(*F*^2^)] = 0.040	Hydrogen site location: difference Fourier map
*wR*(*F*^2^) = 0.116	H-atom parameters constrained
*S* = 1.07	*w* = 1/[σ^2^(*F*_o_^2^) + (0.0582*P*)^2^ + 0.9815*P*] where *P* = (*F*_o_^2^ + 2*F*_c_^2^)/3
1758 reflections	(Δ/σ)_max_ < 0.001
101 parameters	Δρ_max_ = 0.42 e Å^−^^3^
0 restraints	Δρ_min_ = −0.26 e Å^−^^3^
27 constraints	

### Special details

**Table:**

Geometry. All e.s.d.'s (except the e.s.d. in the dihedral angle between two l.s. planes) are estimated using the full covariance matrix. The cell e.s.d.'s are taken into account individually in the estimation of e.s.d.'s in distances, angles and torsion angles; correlations between e.s.d.'s in cell parameters are only used when they are defined by crystal symmetry. An approximate (isotropic) treatment of cell e.s.d.'s is used for estimating e.s.d.'s involving l.s. planes.
Refinement. Refinement of *F*^2^ against ALL reflections. The weighted *R*-factor *wR* and goodness of fit *S* are based on *F*^2^, conventional *R*-factors *R* are based on *F*, with *F* set to zero for negative *F*^2^. The threshold expression of *F*^2^ > σ(*F*^2^) is used only for calculating *R*-factors(gt) *etc*. and is not relevant to the choice of reflections for refinement. *R*-factors based on *F*^2^ are statistically about twice as large as those based on *F*, and *R*- factors based on ALL data will be even larger.

### Fractional atomic coordinates and isotropic or equivalent isotropic displacement parameters (Å^2^)

**Table:**

	*x*	*y*	*z*	*U*_iso_*/*U*_eq_	
S1	0.15096 (4)	0.07607 (7)	0.36393 (5)	0.04497 (19)	
N1	0.16911 (15)	0.3872 (3)	0.27877 (19)	0.0558 (5)	
N2	0.15595 (18)	0.5580 (3)	0.2902 (2)	0.0697 (6)	
N3	0.12550 (16)	0.5769 (3)	0.3536 (2)	0.0652 (6)	
N4	0.11760 (12)	0.4145 (2)	0.38467 (16)	0.0452 (4)	
C1	−0.07348 (14)	−0.1014 (3)	−0.03169 (19)	0.0413 (5)	
H1	−0.1225	−0.1697	−0.0523	0.050*	
C2	0.00541 (14)	−0.1013 (3)	0.08832 (19)	0.0413 (4)	
H2	0.0087	−0.1696	0.1474	0.050*	
C3	0.07963 (13)	0.0003 (3)	0.12106 (17)	0.0384 (4)	
C4	0.16597 (14)	−0.0016 (3)	0.25088 (19)	0.0444 (5)	
H4A	0.2107	0.0712	0.2549	0.053*	
H4B	0.1894	−0.1202	0.2718	0.053*	
C5	0.14563 (12)	0.2996 (3)	0.33927 (16)	0.0390 (4)	
C6	0.08800 (19)	0.3838 (4)	0.4606 (3)	0.0640 (7)	
H6A	0.0387	0.3017	0.4203	0.096*	
H6B	0.0683	0.4921	0.4731	0.096*	
H6C	0.1375	0.3371	0.5385	0.096*	

### Atomic displacement parameters (Å^2^)

**Table:**

	*U*^11^	*U*^22^	*U*^33^	*U*^12^	*U*^13^	*U*^23^
S1	0.0542 (3)	0.0439 (3)	0.0376 (3)	−0.0049 (2)	0.0280 (2)	−0.0001 (2)
N1	0.0711 (13)	0.0491 (11)	0.0643 (12)	−0.0057 (9)	0.0497 (11)	0.0038 (9)
N2	0.0895 (17)	0.0488 (12)	0.0893 (17)	−0.0011 (11)	0.0636 (15)	0.0105 (11)
N3	0.0751 (14)	0.0451 (12)	0.0819 (16)	0.0064 (10)	0.0507 (14)	0.0054 (10)
N4	0.0409 (9)	0.0459 (10)	0.0497 (10)	0.0017 (7)	0.0278 (8)	0.0002 (8)
C1	0.0418 (10)	0.0438 (11)	0.0449 (11)	−0.0055 (8)	0.0296 (9)	−0.0073 (8)
C2	0.0474 (11)	0.0421 (11)	0.0411 (10)	−0.0026 (9)	0.0303 (9)	−0.0021 (8)
C3	0.0387 (10)	0.0404 (10)	0.0391 (10)	−0.0002 (8)	0.0250 (8)	−0.0078 (8)
C4	0.0408 (10)	0.0467 (12)	0.0445 (11)	0.0022 (9)	0.0250 (9)	−0.0040 (9)
C5	0.0336 (9)	0.0449 (11)	0.0335 (9)	−0.0040 (8)	0.0176 (8)	−0.0014 (8)
C6	0.0681 (16)	0.0732 (17)	0.0717 (17)	−0.0009 (13)	0.0532 (15)	−0.0078 (13)

### Geometric parameters (Å, °)

**Table:**

S1—C5	1.732 (2)	C1—H1	0.9300
S1—C4	1.821 (2)	C2—C3	1.394 (3)
N1—C5	1.321 (3)	C2—H2	0.9300
N1—N2	1.353 (3)	C3—C1^i^	1.388 (3)
N2—N3	1.291 (3)	C3—C4	1.509 (3)
N3—N4	1.347 (3)	C4—H4A	0.9700
N4—C5	1.344 (3)	C4—H4B	0.9700
N4—C6	1.450 (3)	C6—H6A	0.9600
C1—C3^i^	1.388 (3)	C6—H6B	0.9600
C1—C2	1.389 (3)	C6—H6C	0.9600
			
C5—S1—C4	100.20 (10)	C3—C4—S1	113.41 (14)
C5—N1—N2	105.50 (19)	C3—C4—H4A	108.9
N3—N2—N1	111.44 (19)	S1—C4—H4A	108.9
N2—N3—N4	106.27 (19)	C3—C4—H4B	108.9
C5—N4—N3	108.20 (18)	S1—C4—H4B	108.9
C5—N4—C6	129.58 (19)	H4A—C4—H4B	107.7
N3—N4—C6	122.13 (19)	N1—C5—N4	108.57 (19)
C3^i^—C1—C2	120.44 (18)	N1—C5—S1	128.22 (17)
C3^i^—C1—H1	119.8	N4—C5—S1	123.19 (15)
C2—C1—H1	119.8	N4—C6—H6A	109.5
C1—C2—C3	120.64 (18)	N4—C6—H6B	109.5
C1—C2—H2	119.7	H6A—C6—H6B	109.5
C3—C2—H2	119.7	N4—C6—H6C	109.5
C1^i^—C3—C2	118.92 (18)	H6A—C6—H6C	109.5
C1^i^—C3—C4	120.31 (18)	H6B—C6—H6C	109.5
C2—C3—C4	120.77 (18)		
			
C5—N1—N2—N3	−0.3 (3)	C5—S1—C4—C3	−76.96 (17)
N1—N2—N3—N4	−0.4 (3)	N2—N1—C5—N4	0.9 (2)
N2—N3—N4—C5	0.9 (3)	N2—N1—C5—S1	−177.38 (17)
N2—N3—N4—C6	177.8 (2)	N3—N4—C5—N1	−1.2 (2)
C3^i^—C1—C2—C3	0.0 (3)	C6—N4—C5—N1	−177.7 (2)
C1—C2—C3—C1^i^	0.0 (3)	N3—N4—C5—S1	177.23 (15)
C1—C2—C3—C4	−178.97 (18)	C6—N4—C5—S1	0.6 (3)
C1^i^—C3—C4—S1	119.20 (18)	C4—S1—C5—N1	−15.1 (2)
C2—C3—C4—S1	−61.8 (2)	C4—S1—C5—N4	166.87 (16)

Symmetry codes: (i) −*x*, −*y*, −*z*.

### Hydrogen-bond geometry (Å, °)

**Table:**

*D*—H···*A*	*D*—H	H···*A*	*D*···*A*	*D*—H···*A*
C4—H4B···N2^ii^	0.97	2.58	3.429 (3)	145
C6—H6A···Cg_benzene_^iii^	0.96	2.82	3.545 (4)	133

Symmetry codes: (ii) *x*, *y*−1, *z*; (iii) −*x*, *y*, −*z*+1/2.
